# Study of Radiation-Induced Defects in *p*-Type Si_1−x_Ge_x_ Diodes before and after Annealing

**DOI:** 10.3390/ma13245684

**Published:** 2020-12-12

**Authors:** Tomas Ceponis, Stanislau Lastovskii, Leonid Makarenko, Jevgenij Pavlov, Kornelijus Pukas, Eugenijus Gaubas

**Affiliations:** 1 Institute of Photonics and Nanotechnology, Vilnius University, Sauletekio ave. 3, LT-10257 Vilnius, Lithuania; jevgenij.pavlov@tmi.vu.lt (J.P.); kornelijus.pukas@tmi.vu.lt (K.P.); eugenijus.gaubas@ff.vu.lt (E.G.); 2 Laboratory of Radiation Effects, Scientific-Practical Materials Research Centre of NAS of Belarus, P. Brovki Str.17, 220072 Minsk, Belarus; lastov@physics.by; 3 Department of Applied Mathematics and Computer Science, Belarusian State University, Independence Ave. 4, 220030 Minsk, Belarus; makleo@mail.ru

**Keywords:** silicon–germanium alloy, electron beam, irradiation-induced defects, DLTS

## Abstract

In this work, electrically active defects of pristine and 5.5 MeV electron irradiated *p*-type silicon–germanium (Si_1−x_Ge_x_)-based diodes were examined by combining regular capacitance deep-level transient spectroscopy (C-DLTS) and Laplace DLTS (L-DLTS) techniques. The *p*-type SiGe alloys with slightly different Ge contents were examined. It was deduced from C-DLTS and L-DLTS spectra that the carbon/oxygen-associated complexes prevailed in the pristine Si_0.949_Ge_0.051_ alloys. Irradiation with 5.5 MeV electrons led to a considerable change in the DLT spectrum containing up to seven spectral peaks due to the introduction of radiation defects. These defects were identified using activation energy values reported in the literature. The double interstitial and oxygen complexes and the vacancy, di-vacancy and tri-vacancy ascribed traps were revealed in the irradiated samples. The interstitial carbon and the metastable as well as stable forms of carbon–oxygen (C_i_O_i_^*^ and C_i_O_i_) complexes were also identified for the electron-irradiated SiGe alloys. It was found that the unstable form of the carbon–oxygen complex became a stable complex in the irradiated and the subsequently annealed (at 125 °C) SiGe samples. The activation energy shifts in the radiation-induced deep traps to lower values were defined when increasing Ge content in the SiGe alloy.

## 1. Introduction

Silicon–germanium alloys are promising materials for the fabrication of photocells and powering space applications [1]. This alloy is also employed in the production of high-frequency heterojunction bipolar transistors for operation in the near THz range [2]. Silicon–germanium provides a novel approach to the formation of high-conversion efficiency and highly scalable thermoelectric materials. Silicon–germanium alloys have recently been reported [3] to function well as lithium-ion battery anodes. This alloy is also prospective for the fabrication of microelectronic and optoelectronic devices such as high-speed temperature sensors, Hall effect transducers and γ-ray detectors [4,5]. Therefore, the spectrum of carrier traps is a desirable characteristic for material quality evaluation.

Silicon–germanium material-based devices are capable of operating in harsh radiation environments [6,7]. Silicon–germanium-based pixel detectors with enhanced radiation tolerance are promising for applications in the future High-Luminosity Large Hadron Collider [8]. However, there are difficulties in growing bulk SiGe single-crystals due to the differences in the physical properties of silicon and germanium such as density and melting temperature. For example, single crystals have only been obtained for alloys containing either 0 < x < 0.1 or 0.85 < x <1 of Ge when using the Czochralski technique. The alloy usually becomes polycrystalline for the other range of Ge content [5,9].

The deep carrier traps affect the characteristics of the semiconductor particle detector [10,11]. Impurities, such as oxygen and carbon, play an important role in the formation of the irradiation-induced deep traps [12,13,14]. Vacancies and their complexes affect the switching properties of the SiGe-based devices [15,16]. In some cases, radiation damage to the Si_1−x_Ge_x_ devices related to the introduction of radiation defects can be “removed” by annealing [17]. This can be implemented by the atomic reconfiguration of the crystal structure during material annealing [18]. However, the radiation defect spectrum in *p*-type Si_1−x_Ge_x_ detectors has been poorly examined. Thus, it is necessary to study the growth and radiation defects in SiGe materials as well as their transformations under annealing.

In this work, the analysis of the electrical characteristics in pristine, electron irradiated and subsequently annealed Si_1−x_Ge_x_ samples with different Ge contents was performed. The routine capacitance deep-level transient spectroscopy (C-DLTS) and Laplace DLTS (L-DLTS) techniques [19,20] were combined to clarify the deep trap spectrum. Correlation of the radiation defect parameters and Ge content in SiGe alloys was examined in the 25–260 K temperature range. Moreover, the annealing caused by transformations of the low-activation energy traps were revealed. Additionally, the growth-associated defects were unveiled only in pristine Si_1−x_Ge_x_ samples containing the largest Ge content values. The traps were identified by analysing the activation energy values reported in the literature. It was revealed that the carbon–oxygen metastable complexes (C_i_O_i_^*^) were transformed into the stable-state complexes (C_i_O_i_) under 125 °C annealing for 15 min of the irradiated samples. 

## 2. Samples and Measurements of Deep Trap Spectra

In this work, the pristine and electron-irradiated Si_1−x_Ge_x_ diodes with an n^+^p structure were examined. The diodes were fabricated using SiGe substrates grown using the Czochralski technique. The diode basis was formed from the *p*-type material (doped with boron), containing either 1%, 1.4% or 5.1% of Ge. For comparison, the diodes made of pure Si used the same (as the SiGe alloys) boron-doping parameters. Irradiation with 5.5 MeV electrons was performed at room temperature using a linear accelerator with electron fluxes of 2 × 10^12^ cm^−^^2^s^−^^1^. The Si as well as the Si_0.99_Ge_0.01_ alloy diodes were irradiated with fluence of 2 × 10^15^ cm^−^^2^. The Si_0.986_Ge_0.014_ and Si_0.949_Ge_0.051_ alloy-based diodes were irradiated with fluences of 5 × 10^13^ cm^−^^2^ and 2 × 10^14^ cm^−^^2^, respectively. The irradiated samples were consequently annealed at 125 °C for 15 min to investigate the changes of DLT spectra under heat treatment.

The DLT spectra were recorded using a commercial HERA-DLTS 1030 instrument (PhysTech GmbH, Moosburg an der Isar, Upper Bavaria, Germany). The DLTS measurements were performed using a routine C-DLTS regime. These DLT spectra were examined in the temperature range of 15–280 K. The majority carrier trap spectra were recorded at reverse bias voltage (*U_R_*) of 3 V and injection pulses (*t_p_*) of 10 ms duration. Each spectrum was analysed by combining correlation functions and the Laplace method.

## 3. Recorded DLT Spectra and Extracted Trap Parameters

**Figure 1:**
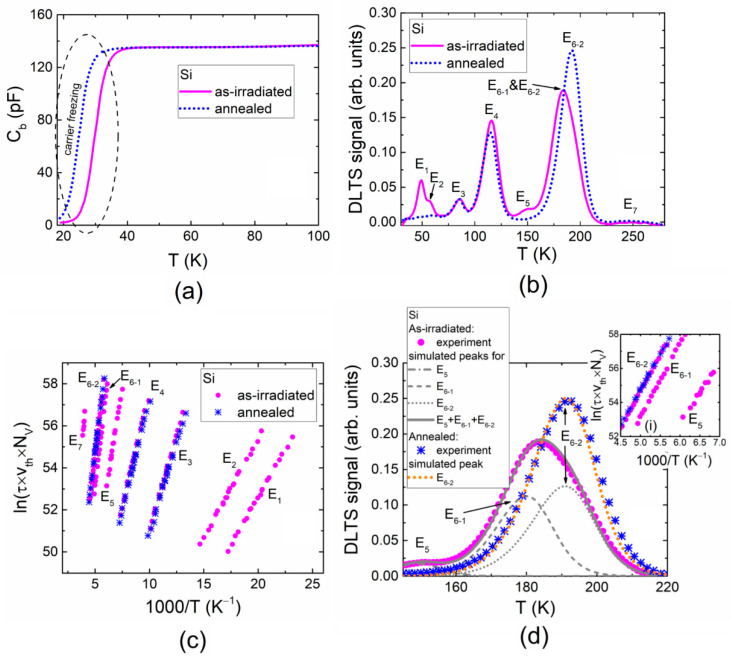
(**a**) The barrier capacitance dependence on temperature (C_b_–T) obtained for 5.5 MeV electron irradiated and subsequently annealed Si samples. (**b**) Deep-level transient (DLT) spectra of the as-irradiated and annealed Si samples. (**c**) Arrhenius plots made for different traps. (**d**) The highlighted spectral range inherent for the E_5_, E_6-1_ and E_6-2_ trap appearances. Inset (i) Arrhenius plots for traps E_6-1_ and E_6-2_. Here, *τ* denotes the carrier lifetime relative to emission; *υ_th_* is the carrier thermal velocity, and *N_V_* stands for the effective density of hole states in the valence band.

Up to seven spectral peaks (assigned to the E_1_–E_7_ traps, as illustrated in Figure 1b) were observed within C-DLT spectra recorded on the 5.5 MeV electron irradiated Si diode when using a fluence of *Φ* = 2 × 10^14^ e/cm^2^. Figure 1a shows the barrier capacitance changes with temperature (C_b_–T) in the 5.5 MeV electron-irradiated and subsequently annealed Si samples. It can be noticed in Figure 1a that an onset within the C_b_–T curves was obtained for the as-irradiated and subsequently annealed Si samples. The shift of the onset may have appeared due to the irradiation- and annealing-induced transformations and density variations of carrier trap species, which caused freezing of carriers within the temperature range under consideration [19,21]. A few peaks (for instance, E_6_) changed their position under annealing relatively to an abscise scale implying the intricate transformation of traps assigned to this spectral peak. Such a spectral range was carefully examined (Figure 1d) using routine and Laplace transform DLTS (L-DLTS). It was clarified that the E_6_ peak can be composed of two peaks E_6-1_ and E_6-2_, just after irradiation. These peaks were ascribed to traps with slightly different activation energies, and their values can be evaluated using Arrhenius plots (as shown within inset (i) for Figure 1d). The concentration and activation energy of traps attributed to the E_6-2_ peak increased after annealing as can be deduced from Figure 1b,d. It is worth mentioning that only the E_6-2_ peak remained after annealing (instead of the E_6-1_ and E_6-2_ as well as E_5_ peaks), and its amplitude was close to the sum of the E_6-1_ and E_6-2_ as well as E_5_ peaks before annealing. This implies that the changes in the E_5_ and E_6-1_ as well as E_6-2_ spectral peaks actually represented transformations of the defects due to the annealing.

The evolution of radiation defects in *p*-type Si introduced by electron beam is rather well understood [18,22]. Thereby, identification of the most resolved traps in Si can be reliably implemented based on activation energy values reported in the literature. Parameters for all the identified Si traps are presented in Table 1.

The trap with the activation energy of 0.080 eV (E_1_ in Table 1) is attributed to the double interstitial and oxygen (I_2_O) complex [18]. The 0.100 eV (E_2_) level can be assigned to a triple vacancy (V_3_) [18]. The origin of the E_3_ trap is not clear, however, it might be related to vacancy (V) [23]. The trap with activation energy of 0.190 eV (E_4_) is associated with V_2_ + V_3_ complex [18,22]. The trap with activation energy of 0.285 eV (E_5_) is associated with a carbon interstitial (C_i_) [18], while the close energy duplet of 0.360 (E_6-1_) and 0.371 (E_6-2_) are attributed to the metastable and stable forms of the carbon–oxygen (C_i_O_i_^*^ and C_i_O_i_) complexes [22,24], respectively. After 15 min annealing at 125 °C, the unstable form of the carbon–oxygen complex seems to become the stable complex according to reactions [22]:

(1) $$C_{i}+O_{i}\overset{reaction}{\to }C_{i}O_{i}^{*}+C_{i}O_{i}\overset{annealing}{\to }C_{i}O_{i}$$

The density of traps ascribed to the stable form of the carbon–oxygen (E_6-2_) complex should hold the density of constituents, represented by the sum of E_5_, E_6-1_ and E_6-2_ DLTS peaks before annealing. The alternative sequence of reactions in formation of the stable E_6-2_ complex would be as follows [22]:

(2) $$I_{2}O\overset{annealing}{\to }I_{2}+O\to O_{i}+C_{i}\overset{annealing}{\to }C_{i}O_{i}$$

The annealing out of E_1_ and E_5_ traps together with an increase in the amplitude of the E_6-2_ peak (Figure 1b) supports the predicted sequences of the reactions denoted in Equations (1) and (2).

**Figure 2:**
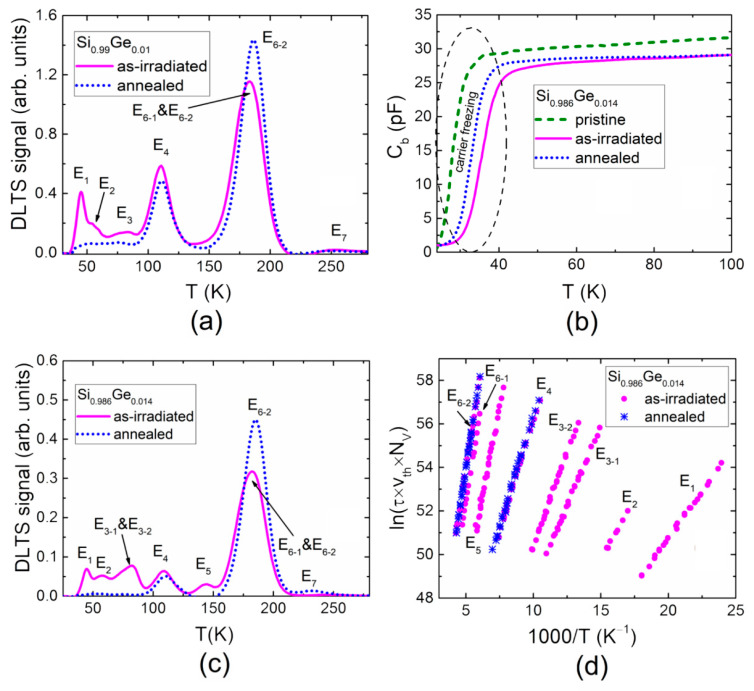
(**a**) DLT spectra recorded on the as-irradiated and annealed Si_0.99_Ge_0.01_ diode samples. (**b**) The barrier capacitance dependence on temperature (C_b_–T) obtained for the pristine, 5.5 MeV electron irradiated and subsequently annealed Si_0.986_Ge_0.014_ samples. (**c**) DLT spectra recorded on the as-irradiated and annealed Si_0.986_Ge_0.014_ diode samples. (**d**) Arrhenius plots composed for different traps.

The slight addition of Ge to get the Si_0.99_Ge_0.01_ alloy should not drastically modify the spectrum of Si radiation defects introduced using the same irradiation conditions (*Φ* = 2 × 10^15^ cm^−2^). Indeed, the structure of the DLT spectrum (illustrated in Figure 2a) and its changes after annealing of the electron-irradiated Si_0.99_Ge_0.01_ diode resembled that obtained in Figure 1. Other parameters for all the revealed traps in the Si_0.99_Ge_0.01_ alloy are presented in Table 2. These results indicate that values of the activation energy, ascribed to the E_1_–E_7_ traps in Figure 1, are shifted to the low-energy range relative to those obtained for the Si of the same type and doping level.

Another slight addition of Ge to obtain the Si_0.986_Ge_0.014_ alloy should further modify the spectrum of Si radiation defects. Figure 2b shows the barrier capacitance changes with temperature (C_b_–T) in the 5.5 MeV electron-irradiated and subsequently annealed Si_0.986_Ge_0.014_ samples. However, together with the onset within the C_b_–T curves, observed in Figure 1a, variations of the slope of the C_b_–T curves can be noticed for the pristine, as-irradiated and subsequently annealed Si_0.986_Ge_0.014_ samples. These changes in the C_b_–T curve onsets and slopes can be ascribed to irradiation and annealing-induced transformations and various trap species density variations that cause freezing of carriers within the temperature range under consideration [19,21]. Indeed, the structure of the DLT spectrum (Figure 2c) and its changes after annealing of the electron irradiated Si_0.986_Ge_0.014_ diode resembles that obtained in Figure 1b. Moreover, it was observed that the E_3_ peak was composed of two peaks E_3-1_ and E_3-2_, as obtained after irradiation of the Si_0.986_Ge_0.014_ alloy diode. The latter (E_3-1_ and E_3-2_) peaks were ascribed to traps with slightly different activation energies, which had values that were determined by using Arrhenius plots (Figure 2d). The E_3-1_ and E_3-2_ peaks disappeared after annealing, thereby indicating the transformation of vacancy related defects.

The L-DLTS technique was additionally employed to separate the E_6-1_ and E_6-2_ traps more precisely. The trap activation energy values were evaluated using Arrhenius plots (Figure 2d). Parameters for all the unveiled traps are presented in Table 2. However, values of the activation energy ascribed to the E_1_–E_7_ traps, illustrated in Figure 2c and listed in Table 2, were close to those extracted from the Si_0.99_Ge_0.01_ spectra (Figure 2a). However, these activation energy values were slightly different from those obtained for the Si diodes. The activation energy values extracted for Si_0.986_Ge_0.014_ diodes were again shifted to the low-energy range relative to those obtained for Si of the same conductivity type.

**Figure 3:**
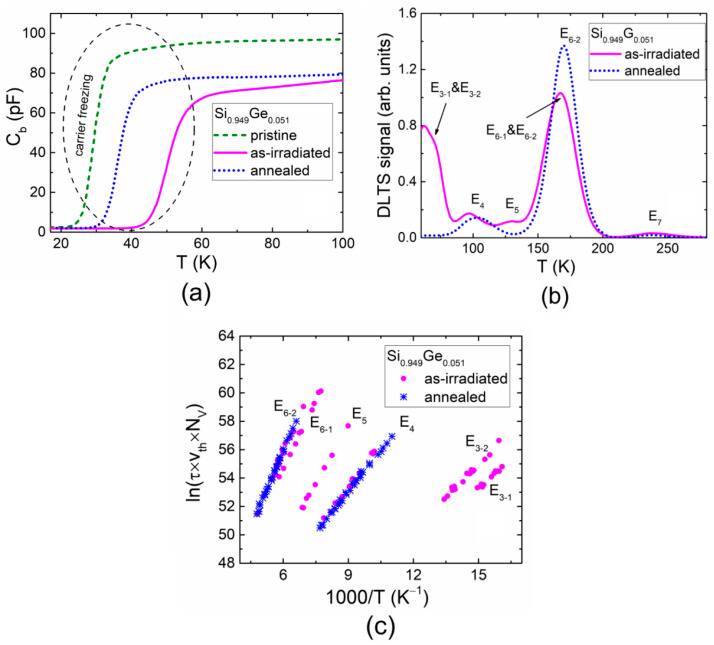
(**a**) The barrier capacitance dependence on temperature (C_b_–T) obtained for pristine, 5.5 MeV electron irradiated and the subsequently annealed Si_0.949_Ge_0.051_ samples. (**b**) DLT spectra recorded on the as-irradiated and annealed Si_0.949_Ge_0.051_ samples. (**c**) Arrhenius plots made for different traps.

The rather different DLTS characteristics (relative to those of pure Si as well as of 1% and 1.4% Ge-containing SiGe alloy) were obtained for the 5.5 MeV electron-irradiated and subsequently annealed Si_0.949_Ge_0.051_ material diodes. Figure 3a illustrates the barrier capacitance changes with temperature (C_b_–T) in the pristine, in the 5.5 MeV electron-irradiated and the subsequently annealed Si_0.949_Ge_0.051_ samples. The change in the slope of the C_b_–T curves was inherent for all the pristine, the as-irradiated and the subsequently annealed samples. Again, the onsets within the C_b_–T curves and slightly different slopes seem to appear due to the irradiation- and annealing-induced transformations and various traps species density variations [19,21].

The L-DLTS method was chosen to separate the traps inherent for low-temperature range (40–80 K) owing to the L-DLTS elevated resolution (up to 2 MeV). The DLT spectra recorded on the as-irradiated and annealed Si_0.949_Ge_0.051_ samples are illustrated in Figure 3b. However, here, the DLT spectra covered the temperature range >50 K. The barrier capacitance of the Si_0.949_Ge_0.051_ diodes vanished in the low temperature range (Figure 3a), and the application of the capacitance DLTS was then impossible [19]. The activation energies of the traps were evaluated using Arrhenius plots (Figure 3c). The spectra with two prevailing peaks, namely, E_4_ and E_6-2_, were again recorded after annealing at 125 °C for 15 min, similar to the regularity observed for the Si_0.986_Ge_0.014_ sample. The DLTS signatures for all the traps observed in Si_0.949_Ge_0.051_ samples are listed in Table 2. It was obtained that the activation energy values in the Si_0.949_Ge_0.051_ alloy were shifted even more to the low-energy range relative to those obtained for the Si, Si_0.99_Ge_0.01_ and Si_0.986_Ge_0.014_ samples.

**Figure 4:**
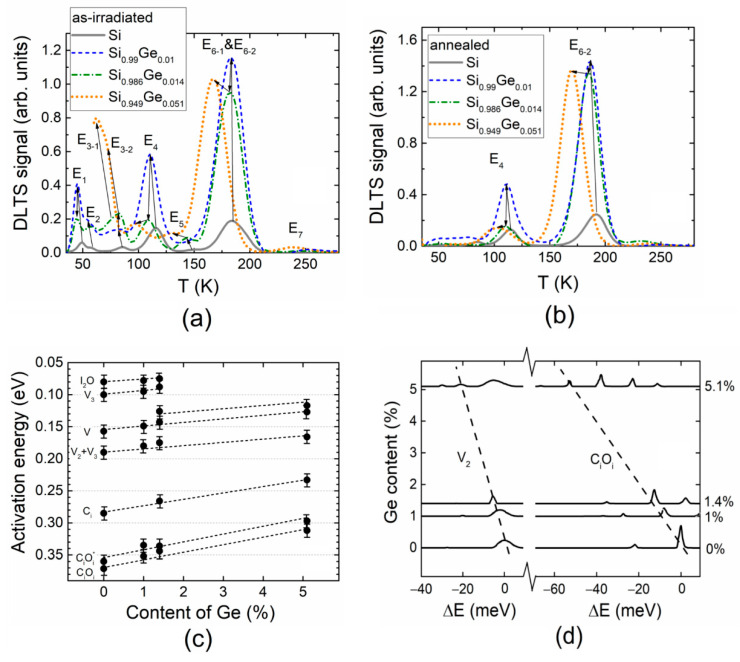
Comparison of the DLT spectra obtained in the as-irradiated (**a**) and annealed (**b**) Si and SiGe alloy samples. (**c**) The tendency of changes in the activation energy values of the radiation-induced traps in Si_1−x_Ge_x_ alloy as a function of Ge content. (**d**) Ge content dependent variations in the Laplace DLT spectra obtained for V_2_ and C_i_O_i_ radiation-induced defects.

The comparison of the DLT spectra obtained in the as-irradiated (a) and annealed (b) Si and SiGe alloy samples is generalized in Figure 4 to clarify the activation energy shifts. 

The tendency of changes in the activation energy values of traps in Si_1__−__x_Ge_x_ alloy as a function of Ge content is sketched in Figure 4c, based on DLT spectroscopy data obtained in this work. Additionally, the Laplace DLT spectra were re-plotted according to the re-calculation procedure described in Reference [26]. These L-DLT spectra are illustrated in Figure 4d to highlight the shifts in energy levels ascribed to V_2_ and C_i_O_i_ defects in the as-irradiated diodes. It can be inferred from Figure 4a,b, that the activation energy values decreased significantly with enhancement of the Ge content in the SiGe alloy, irrespective of the irradiation and annealing procedures. This activation energy variation was also independent of the DLTS peak amplitude changes. These results can be understood as an indication that the levels moved closer to the valance band [12,27]. The peak shifts to the higher energy range with an increase in the Ge content was obtained for n-type SiGe alloys [28,29,30] conversely to those investigated in this work—*p*-type SiGe alloys. This effect can be explained either through an occupation of the radiation defect core by Ge atoms in the SiGe alloy due to the lattice parameter change or via lattice bond length variations which affect the conduction and valence band parameters of the SiGe alloy [12]. The opposite tendency within the observed DLTS peak shifts for the *p*-type and *n*-type SiGe alloys might be alternatively explained through the shifts of the Fermi level and the consequent filling of the radiation defect states.

## 4. Summary

The deep trap spectra in the pristine, 5.5 MeV electron irradiated and the 125 °C annealed *p*-type SiGe alloys with slightly different Ge content were examined. It was deduced from C-DLTS and L-DLTS spectra that the carbon/oxygen associated complexes prevailed in the pristine Si_0.949_Ge_0.051_ alloys. Irradiation with 5.5 MeV electrons led to considerable change in the DLT spectrum containing up to seven spectral peaks due to the introduction of the radiation defects. These defects have been identified using activation energy values reported in the literature. The double interstitial and oxygen (I_2_O) complexes and the vacancy, di-vacancy and tri-vacancy ascribed traps were revealed in the irradiated samples. The interstitial carbon and the metastable as well as stable forms of the carbon–oxygen (C_i_O_i_^*^ and C_i_O_i_) complexes were also identified for the irradiated SiGe alloys. It was found that the carbon–oxygen metastable complexes (C_i_O_i_^*^) were transformed into stable-state complexes (C_i_O_i_) under 125 °C annealing for 15 min of the irradiated samples. It was determined that the activation energy shifts of radiation-induced deep traps to low values were defined by an increase in the Ge content of the SiGe alloy.

## Figures and Tables

**Table 1:** Traps revealed for the 5.5 MeV electron as-irradiated and subsequently annealed Si sample.

Sample	As-Irradiated			Subsequently Annealed			Origin of Defect According to [Reference]
DLTS Peak	Activation Energy (eV)	Capture Cross-Section (cm^2^)	Density of Traps (cm^−^^3^)	Activation Energy (eV)	Capture Cross-Section (cm^2^)	Density of Traps (cm^−^^3^)	
E_1_	0.080	1.36 × 10^−15^	6.40 × 10^12^				I_2_O [18]
E_2_	0.100	1.04 × 10^−14^	3.24 × 10^12^				V_3_ [18]
E_3_	0.157	5.72 × 10^−15^	3.36 × 10^12^	0.150	2.01 × 10^−15^	3.55 × 10^12^	V-related [23]
E_4_	0.190	3.60 × 10^−16^	1.55 × 10^13^	0.185	1.88 × 10^−16^	1.36 × 10^13^	V_2_ + V_3_ [18,22]
E_5_	0.285	4.69 × 10^−15^	1.61 × 10^12^				C_i_ [18,22]
E_6-1_	0.360	9.32 × 10^−15^	1.22 × 10^13^				C_i_O_i_^*^ [22]
E_6-2_	0.371	4.25 × 10^−15^	1.41 × 10^13^	0.370	3.90 × 10^−15^	2.66 × 10^13^	C_i_O_i_ [22]
E_7_	0.500	4.46 × 10^−15^	6.07 × 10^11^				I-C_i_/I-B_i_ [25]

**Table 2:** Summary of traps revealed for the as-irradiated and subsequently annealed SiGe samples with different Ge content.

Sample	As-Irradiated			Subsequently Annealed		
DLTS Peak	Activation Energy (eV)	Capture Cross-Section (cm^2^)	Density of Traps (cm^−3^)	Activation Energy (eV)	Capture Cross-Section (cm^2^)	Density of Traps (cm^−3^)
**Si_0.99_Ge_0.01_**						
E_1_	0.078	5.83 × 10^−15^	3.00 × 10^13^			
E_2_	0.095	8.70 × 10^−15^	1.07 × 10^13^			
E_3_	0.149	2.86 × 10^−15^	1.08 × 10^13^			
E_4_	0.180	3.21 × 10^−16^	4.79 × 10^13^	0.180	3.07 × 10^−16^	3.80 × 10^13^
E_6-1_	0.335	2.68 × 10^−15^	3.51 × 10^13^			
E_6-2_	0.352	2.09 × 10^−15^	6.13 × 10^13^	0.351	2.24 × 10^−15^	1.11 × 10^14^
E_7_	0.542	2.68 × 10^−14^	1.40 × 10^12^	0.523	2.49 × 10^−14^	7.16 × 10^11^
**Si_0.986_Ge_0.014_**						
E_1_	0.075	3.84 × 10^−15^	1.18 × 10^12^			
E_2_	0.088	1.05 × 10^−15^	8.05 × 10^11^			
E_31_	0.126	1.98 × 10^−15^	4.65 × 10^11^			
E_32_	0.143	2.14 × 10^−15^	6.02 × 10^11^			
E_4_	0.175	2.78 × 10^−16^	1.09 × 10^12^	0.175	2.03 × 10^−16^	8.87 × 10^11^
E_5_	0.266	2.99 × 10^−15^	5.03 × 10^11^			
E_6-1_	0.336	4.94 × 10^−15^	2.60 × 10^12^			
E_6-2_	0.344	1.81 × 10^−15^	4.25 × 10^12^	0.346	5.56 × 10^−15^	7.09 × 10^12^
E_7_				0.585	6.12 × 10^−13^	1.10 × 10^11^
**Si_0.949_Ge_0.051_**						
E_31_	0.117	5.42 × 10^−^^15^	1.27 × 10^13^			
E_32_	0.127	5.09 × 10^−^^15^	1.05 × 10^13^			
E_4_	0.170	2.00 × 10^−^^16^	2.50 × 10^12^	0.166	3.03 × 10^−^^16^	2.37 × 10^12^
E_5_	0.233	3.38 × 10^−^^15^	1.52 × 10^12^			
E_6-1_	0.297	1.94 × 10^−^^15^	7.57 × 10^12^			
E_6-2_	0.312	1.12 × 10^−^^15^	1.13 × 10^13^	0.312	1.32 × 10^−^^15^	2.13 × 10^13^
E_7_	0.585	6.12 × 10^−^^13^	1.10 × 10^11^			
